# Serological Evaluation of Antibody Titers After Vaccination Against COVID-19 in 18-44-Year-Old Individuals at a Tertiary Care Center

**DOI:** 10.7759/cureus.40543

**Published:** 2023-06-16

**Authors:** Rachita Nanda, Prishni Gupta, Anjan Kumar Giri, Suprava Patel, Seema Shah, Eli Mohapatra

**Affiliations:** 1 Biochemistry, All India Institute of Medical Sciences, Raipur, Raipur, IND; 2 Community and Family Medicine, All India Institute of Medical Sciences, Raipur, Raipur, IND

**Keywords:** immune response, vaccines, t-cell response, neutralization, igg2, antibody

## Abstract

Background

The evaluation of the effectiveness of the vaccines (ChAdOx1-nCOV; Covishield and BBV-152; Covaxin) against coronavirus disease 2019 (COVID-19) is necessary to assess their efficacy. Because most antibodies that neutralize the coronavirus are directed against the receptor binding domain within the spike protein of the virus, these antibodies serve as markers for viral neutralizers and, in turn, for vaccine response. The present study aimed to evaluate the anti-neutralizing antibody (receptor binding domain (RBD)) and immunoglobulin G2 (IgG2) titers following the completion of the vaccination schedule (both vaccines) against severe acute respiratory syndrome coronavirus 2 (SARS-CoV-2).

Methodology

In this longitudinal prospective study, conducted in a tertiary care center, 30 sequentially (two doses) vaccinated study participants between the ages of 18 and 44 years were sampled for estimation of anti-RBD antibody titer and IgG2. All statistical analysis was done using SPSS version 20 (IBM Corp., Armonk, NY, USA). P-values less than 0.05 were considered significant.

Results

There was a statistically significant increase in the neutralizing antibody titer after one month of the second dose (z = -4.597, p < 0.001), while a significant decrease was seen in the IgG2 levels (z = -3.075, p = 0.002). The results showed a significant neutralizing effect of the vaccines being used, with Covishield being more effective than Covaxin. The levels of neutralizing antibodies were independent of all demographic variables such as age, sex, and body mass index.

Conclusions

This study evaluating the efficacy of the two vaccines, namely, Covishield and Covaxin, is the first of its kind in the state of Chhattisgarh. The results of this study are similar to previous studies conducted in India and outside India, concluding that Covishield is a more effective vaccine.

## Introduction

The world was hit by a catastrophe when a novel coronavirus spread like fire across the continents. A worldwide lockdown ensued to contain the severe acute respiratory syndrome coronavirus (SARS-CoV-2) or the coronavirus disease 2019 (COVID-19) pandemic. Yet, the number of COVID-19-positive cases reached a record 628,694,934 with 6,576,088 deaths according to the World Health Organization (WHO) database on November 4th, 2022 [1]. The heavy caseload and tremendous economic loss led to a gold race for immune kinetic studies followed by vaccine manufacturing at a pace never seen before [2].

In the search for the right vaccine, it was observed that the coronavirus spike protein (S protein) could be the perfect target for the vaccines, especially the receptor binding domain (RBD) [3]. The neutralizing antibodies (NAbs) when bound to this domain oppose the conformational change mandatory for the attachment of the virus to the angiotensin-converting enzyme 2 (ACE2) receptors, disabling entry into the host cell. Thus, the evaluation of the efficacy of these vaccines revolved around their potential to generate humoral immunity and neutralizing abilities [4].

As part of the vaccination revolution, India started its first phase of vaccine administration in January 2021. The Serum Institute of India manufactured AZD1222-ChAdOx1-S (Covishield) and Bharat Biotech, India (in collaboration with the Indian Council of Medical Research) produced BBV152 (Covaxin) [5]. The Covishield was a recombinant, replication-deficient chimpanzee adenovirus vector simulating the SARS-CoV-2 spike (S) glycoprotein, and the Covaxin was made by the whole virion SARS-CoV-2 vaccine strain NIV-2020-770 (spike variant Asp614Gly) inactivated with β-propiolactone [6].

The post-infection immune response in COVID-19 patients has been well studied and characterized now; however, the post-vaccination immunity development still has scope for understanding and judgment [7]. Both humoral immunity provided by B-cell-derived antibodies and cell-mediated immunity driven by the CD4+ and CD8+ T-cells play their roles. The anti-S protein antibodies and S-protein targeted NAbs show a positive correlation with the severity of the disease [8]. Thus, it was concluded that vaccines against COVID-19 had to elicit NAbs and, in turn, these antibodies could serve as markers for viral neutralizers and for vaccine response [9]. T-cells are also important mediators in host response in viral infections by destroying infected cells, promoting B cell function and antibody responses, and reducing the risk of vaccine-induced exaggerated disease conditions [10,11]. Considering how the cellular response can also serve as good a indicator of the immune response, T-cell response measurement could complement tests to assess vaccine efficacy where antibody function is unaccounted for. This study aimed to evaluate the anti-neutralizing antibody and immunoglobulin G2 (IgG2) titers following the completion of the vaccination schedule (both vaccines) against SARS-CoV-2.

## Materials and methods

A longitudinal, prospective study was conducted in the Department of Biochemistry of a tertiary care center (All India Institute of Medical Sciences, Raipur, Chhattisgarh, India) with approval from the institute ethics committee (IEC number: AIIMSRPR/IEC/2021/883) in compliance with the Declaration of Helsinki. A total of 123 participants were recruited after obtaining informed consent. Serum samples were collected after the first dose, and patients were asked to report after one month of the second dose of the COVID-19 vaccine. Out of the 123, only 30 reported for follow-up, and serum was collected. Both sets of samples were analyzed for anti-RBD antibody and IgG2 levels.

Anti-RBD antibody was estimated by the ADVIA Centaur COV2G assay which is a sandwich immunoassay using indirect chemiluminescent technology and reported as index/mL. IgG2 was measured by enzyme-linked immunosorbent assay (Shanghai Coon Koon Biotech Co. Ltd. Human Immunoglobulin G2 (IgG2) Enzyme-Linked Immunosorbent Assay Kit) reported as ng/mL.

Data collected were represented and statistically analyzed using SPSS version 20 (IBM Corp., Armonk, NY, USA). Categorical data were represented as frequency and percentage. Continuous data were checked for normality. Normally distributed data were presented as mean ± SD. Non-normally distributed data were represented as median (interquartile range). Continuous independent data were analyzed by the t-test and analysis of variance (ANOVA) for parametric distributed data, while non-parametric distributed data were compared by the Mann-Whitney U test and Kruskal-Wallis test. Serial antibody titers were compared by Wilcoxon signed rank test for paired data. Categorical data were analyzed by the chi-square test of association. Bivariate Spearman rho was applied to obtain the correlation coefficients. Multivariate linear and binomial regression models were analyzed for continuous and binomial categorical dependents, respectively. P-values <0.05 were considered statistically significant. Box plots, scatter plots, and trend lines were drawn as necessary.

## Results

The present study recruited 123 volunteers to participate in the study, of whom 98 were administered Covishield and 25 were vaccinated with Covaxin. The majority of the volunteers were males (72) with a mean age of 30.50 ± 7.03 years. Anti-RBD antibody and IgG2 levels were estimated in these volunteers. The median level of the serum anti-RBD antibody was found to be 1.310 (5.72) and serum IgG2 was found to be 304.72 (168.96). Out of the 123 subjects, only 30 reported for follow-up, and a second serum sample was collected. There was no significant difference in levels of the NAbs and IgG2 after the first dose of vaccination among the 30 volunteers who reported for the second sample (NAb = 1.20 (6.50); IgG2 = 302.46 (64.89)) and the 73 volunteers who dropped out of the study (NAb = 1.56 (5.03); IgG2 = 305.28 (52.37)).

The data from the remaining participants were further analyzed. The clinical-demographical data of these 30 patients are presented in Table 1.

**Table 1:** Demographic, clinical, and vaccination details of the study group. *: Normally distributed data are represented as mean ± SD. Non-normally distributed data are represented as median (IQR). BMI: body mass index; Temp: temperature; BP: blood pressure; n: frequency; Ab: antibody

Demographical variables	Descriptive frequency	
Age (years) (mean ± SD)	32.20 ± 8.27	
Height (cm) (mean ± SD)	159.84 ± 7.35	
Weight (kg) (mean ± SD)	62.19 ± 12.92	
BMI (kg/m^2^) (mean ± SD)	24. 28 ± 4.36	
Temp (F) (mean ± SD)	97.08 ± 0.90	
Systolic BP (mm/Hg) (mean ± SD)	122.87 ± 5.00	
Diastolic BP (mm/Hg) (mean ± SD)	81.07 ± 4.63	
Pulse (beats/minute) (mean ± SD)	80.27 ± 7.36	
Gender (n)	Male	Female
	17	13
Clinical parameters (n)	Yes	No
Medical History	4	26
Previous COVID infection	14	16
Symptoms of COVID	5	9
Vaccine details (n)	Covishield	Covaxin
	14	16
Adverse effects (n)	Yes	No
After the first dose	1	29
After the second dose	4	26
Antibody levels after vaccination after the first dose (median (IQR))		
Anti-RBD Ab titers (index unit)	1.56 (5.03)	
IgG2 (ng/mL)	305.28 (52.37)	
Antibody levels after vaccination after the second dose (median (IQR))		
Anti-RBD Ab titers (index unit)	29.56 (81.97)	
IgG2 (ng/mL)	290.26 (38.35)	
Seroconversion (n)	Yes	No
	23	7

Covishield vaccine was administered to 46.7% and Covaxin was given to 53.3% of volunteers. The majority of the patients did not show any immediate or remote adverse events. There was a significant rise in the median levels of the anti-RBD antibody after one month of the second dose of the vaccine, with seropositivity seen in 76.7%. The median levels of IgG2 showed a decrease in these volunteers after a month of administration of the second dose of the vaccine.

**Figure 1:**
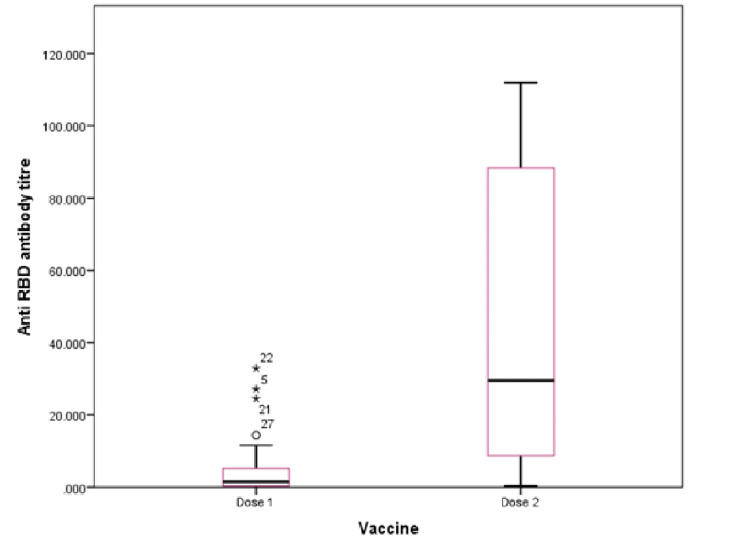
Box plot showing the median levels of the neutralizing antibodies. RBD: receptor binding domain

Wilcoxon test for paired non-parametric data, when applied to compare the antibody titers post the first and second dose of the vaccine, showed that there was a marked rise in the anti-RBD antibody after one month of the second dose of the vaccine. Irrespective of the vaccine administered, the rise in the antibody titer was statistically significant (Figure 1).

**Figure 2:**
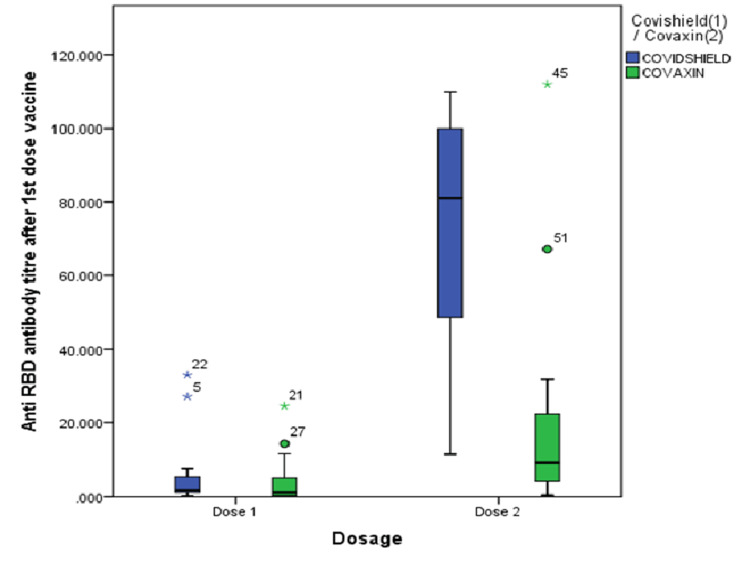
Box plot showing the median levels of the neutralizing antibodies after the first and second doses of Covishield and Covaxin. RBD: receptor binding domain

However, the increase was more marked in the volunteers vaccinated with Covishield (Figure 2).

**Figure 3:**
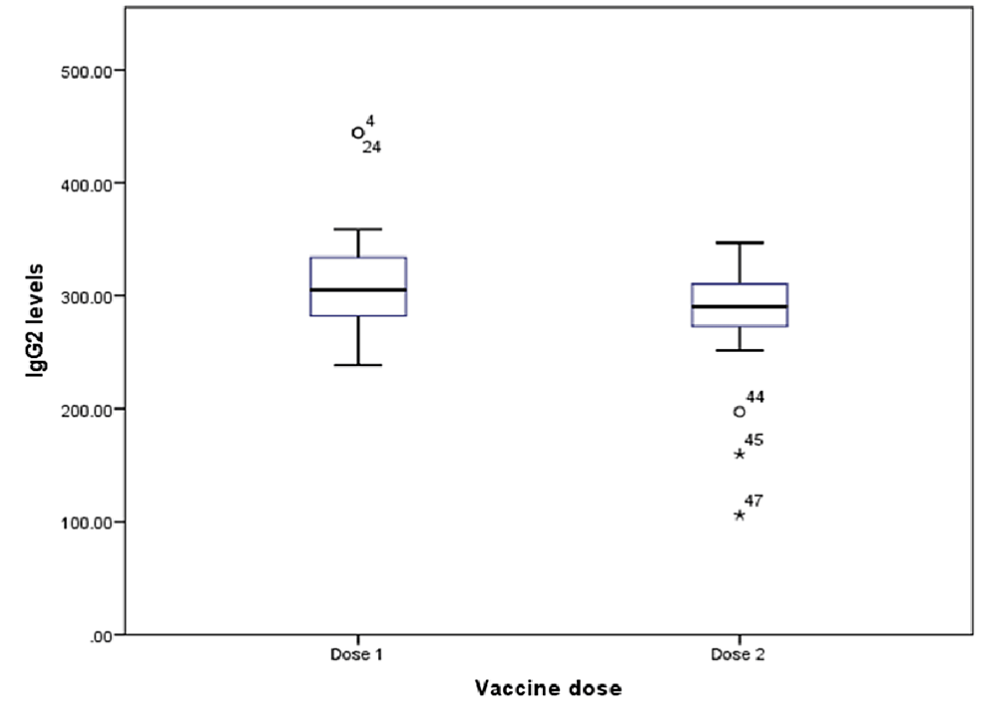
Box plot showing the median levels of IgG2. IgG2: immunoglobulin G2

The IgG2 median levels similarly showed a significant decrease in volunteers vaccinated with Covishield (Figure 3).

In volunteers vaccinated with Covaxin, the difference was not significant (Table 2).

**Table 2:** Comparison of serial antibody titers after each dose of vaccination. *: Non-parametric data represented as median (IQR) and compared by Wilcoxon signed rank paired sample test. RBD: receptor binding domain; Ab: antibody; IgG2: immunoglobulin G2

Vaccine	Antibody	After the first dose, median (IR)	After the second dose, median (IR)	Wilcoxon test	
				Z	P-value
Covishield, n = 14	Anti-RBD Ab	1.695 (4.847)	81.05 (55.94)	-3.296	0.001
	IgG2	324.632 (42.825)	296.49 (43.575)	-2.480	0.013
Covaxin, n = 16	Anti-RBD Ab	1.080 (5.070)	9.070 (22.238)	-3.051	0.002
	IgG2	294.62 (31.809)	281.40 (46.474)	-1.862	0.063
Overall	Anti-RBD Ab	1.565 (5.033)	29.565 (81.965)	-4.597	<0.001
	IgG2	305.285 (52.373)	290.260 (38.350)	-3.075	0.002

**Figure 4:**
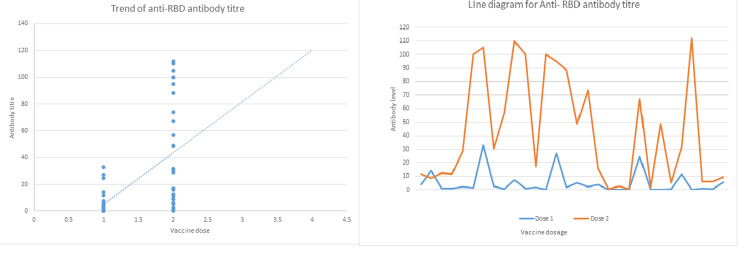
Scatter plot and line diagram plotted for each patient showing the levels of the NAbs after the first and second dose of the vaccine. NAbs: neutralizing antibodies; RBD: receptor binding domain

**Figure 5:**
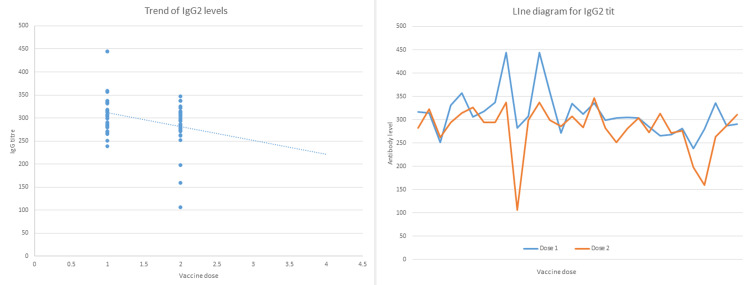
Scatter plot and line diagram plotted for each patient showing the levels of IgG2 after the first and second dose of the vaccine. IgG2: immunoglobulin G2

The anti-RBD antibody and the IgG2 titers after each of the vaccine doses were plotted for each patient to observe their rising and falling trends over time. Figure 4 and Figure 5 are the scatter plots and line diagrams for anti-RBD antibody titers and levels of IgG2 levels in study patients, respectively.

The efficacies of both vaccines were assessed by comparing the levels of NAbs and IgG2 and the percentage of seroconverted volunteers. Although both vaccines showed a rise in the anti-RBD antibody levels, the rise was much higher with Covishield. As a complement to this, the seroconversion was 100% in these volunteers while the seropositivity was 56.25% (p = 0.005) in the Covaxin-administered group. The occurrence of adverse events was also higher in the latter group pointing to a more fruitful effect of the Covishield vaccine (Table 3).

**Table 3:** Comparison between the two vaccines available against COVID-19. *: Normally distributed data represented as mean ± SD and compared by the t-test. Non-normally distributed data represented as median (IQR) and compared by the Mann-Whitney U test. The chi-square test was done to determine the association between categorical data. RBD: receptor binding domain; Ab: antibody; IgG2: immunoglobulin G2

Parameters	Covishield (n = 14)	Covaxin (n = 16)	P-value
Age (mean ± SD)	34.79 ± 6.93	29.94 ± 8.87	0.105
Adverse effects after the first dose (n)	0	1	0.0341
Adverse effects after the second dose (n)	0	4	0.044
Seropositivity (n)	14	9	0.005
Antibody titers for vaccination (median (IR))			
Anti-RBD Ab titer after the first dose	1.695 (4.847)	1.08 (5.07)	0.371
Anti-RBD Ab titer after the second dose	81.05 (55.94)	9.07 (22.24)	<0.001
Anti-RBD Ab titer rise	23.24 (47.15)	29.53 (91.83)	0.618
IgG2 levels after the first dose	324.63 (42.82)	294.62 (31.809)	0.025
IgG2 levels after the second dose	296. 49 (43.57)	281.40 (46.47)	0.067
Decrease in the IgG2 levels	7.84 (48.92)	28.09 (96.29)	0.081

**Figure 6:**
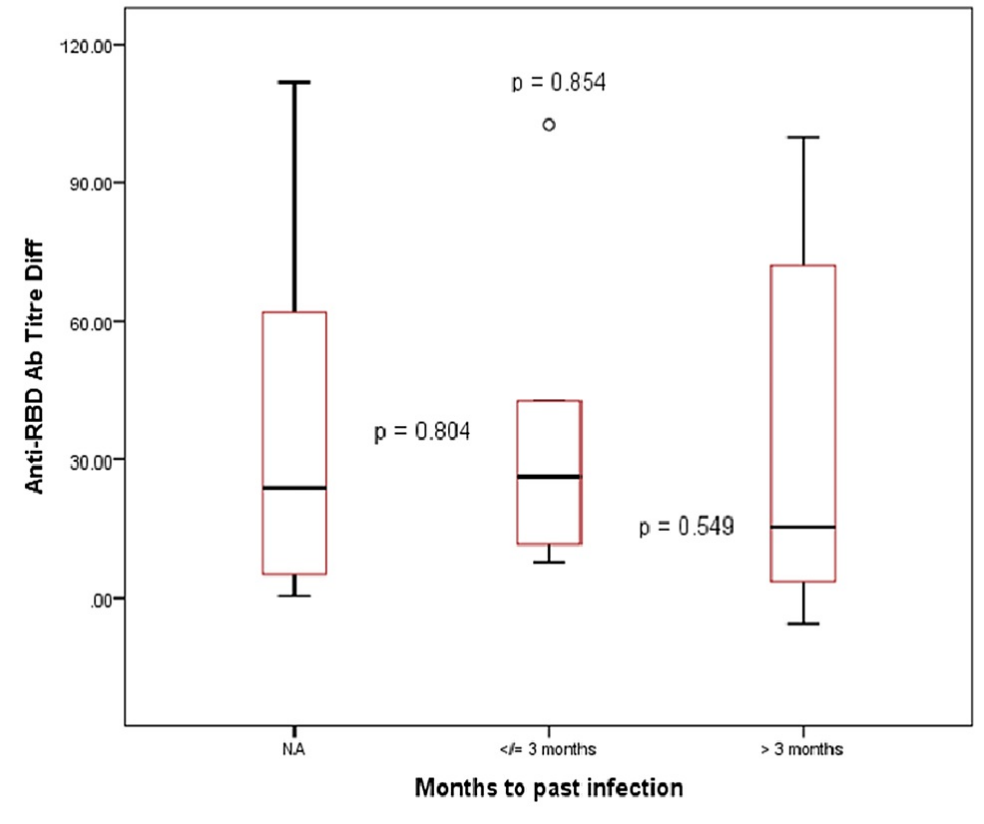
Box plot showing the median levels of NAbs in volunteers with and without previous infection. NAbs: neutralizing antibodies; NA: not infected previously; RBD: receptor binding domain

Previous exposure to this alarming infection led to a significantly increased NAb level immediately after the first dose; however, the antibody titer after an interval of one month of the second dose was similar to the unexposed group. When compared between groups, the volunteers who suffered from COVID-19 in the past three months of recruitment showed a higher median level of NAbs than the group with exposure time more than three months apart. No significant association was observed between seropositivity and a previous COVID-19 infection (Table 4, Table 5, and Figure 6).

**Table 4:** Antibody titers and seropositivity in patients with previous exposure to COVID-19. *: Non-parametric data represented as median (IQR) and compared by the Mann-Whitney U test. Categorical data were analyzed by the chi-square association test. RBD: receptor binding domain; Ab: antibody; IgG2: immunoglobulin G2

Antibody titers after vaccination (Median (IR))	Previous COVID-19 infection		Mann-Whitney U test
	No (n= 16)	Yes (n= 14)	p
Anti-RBD Ab titer after the first dose	0.83 (2.27)	3.18 (8.06)	0.031
Anti-RBD Ab titer after the second dose	31.00 (75.12)	23.05 (90.75)	0.519
Anti-RBD Ab titer rise	49.93 (82.92)	5.26 (41.83)	0.022
IgG2 levels after the first dose	294.88 (57.55)	315.46 (44.52)	0.044
IgG2 levels after the second dose	285.98 (41.68)	296.49 (37.26)	0.406
Decrease in the IgG2 levels	31.78 (60.09)	7.85 (49.46)	0.228
Seropositivity			Chi-square test p-value
No	4	3	0.818
Yes	12	11	

**Table 5:** Comparison between volunteer groups with previous COVID-19 infection. *: Kruskal Wallis test applied to compare groups with non-parametric distribution. RBD: receptor binding domain; Ab: antibody; IgG2: immunoglobulin G2

Antibody titers after vaccination (median (IR))	Previous COVID-19 infection			Kruskal-Wallis test
	NA (n = 16)	≤3 (n = 5)	>3 (n = 9)	P-value
Anti-RBD Ab titer after the first dose	0.83 (2.27)	4.16 (12.83)	1.74 (9.34)	0.049
Anti-RBD Ab titer after the second dose	31.00 (75.12)	28.84 (74.95)	17.26 (92.54)	0.765
Anti-RBD Ab titer rise	23.80 (59.99)	26.36 (62.96)	15.52 (82.46)	0.053
IgG2 levels after the first dose	294.88 (57.55)	298.98 (53.25)	318.40 (41.36)	0.064
IgG2 levels after the second dose	285.98 (41.684)	281.74 (108.83)	298.66 (42.05)	0.152
Decrease in the IgG2 levels	15.70 (52.11)	34.74 (95.21)	23.65 (76.44)	0.515

Regression analysis with all the variates predicted that only the type of vaccine affected the antibody levels and seroconversion significantly (R^2^ = 0.317, F = 12.323, p = 0.002). The Hosmer and Lemeshow p-value of 0.408 showed that the predictive model constructed utilizing sex, age, body mass index, previous infection, and type of vaccine was a good fit model, accurately analyzing 76.7% cases of seropositivity with a positive predictive value of 91.3%. Each of the clinical-demographical variables, when analyzed individually, did not show a significant change in the antibody levels and seropositivity, proving that the vaccine efficacy was independent of all the variables.

## Discussion

In 2019, a severe pneumonia breakdown in Wuhan city of China became a new global public health crisis. While the whole of the seafood market was shut down, a surveillance system was started, and on the seventh of January 2020, the infective organism was identified as a coronavirus, sharing similarities with the bat coronavirus and SARS-CoV. As the fatalities started being reported, the virus spread to the neighboring countries as well. Soon, parts of China were locked down, and with human-to-human transmission by droplets, every country pulled the shutters to their airports [12]. This novel virus disease was named COVID-19 and it spread like fire across continents, meshing through barriers, and the mortality reached millions, affecting the major economies of the world. Like all viral infections, the vaccine for this misfortune was sought, as the only remedy to battle the pandemic.

The coronavirus, as the name suggests, appeared crown-like under the electron microscope. With a single-stranded positive RNA as the genetic material, it is the largest known genome among all RNA viruses. The virus structure consists of different layers, starting from the exterior spike protein, membrane protein, and envelope protein layer to the interior nucleocapsid containing the viral genome [13]. According to the structural units, various types of vaccines could be formulated, such as whole-inactivated virus vaccines, live-attenuated virus vaccines, viral vector vaccines, subunit vaccines, and DNA and RNA vaccines [14]. With the urgency to develop the most effective vaccine against the ongoing pandemic, the world’s fastest vaccines were developed.

In June 2020, China brought its first vaccine named CanSino for use in military personnel, and two other inactivated vaccines for emergency high-risk cases [15]. Russia was next to develop Sputnik V which was also exported for phase III clinical trial [16]. The FDA then approved the Pfizer-BioNtech, an mRNA vaccine (BNT162b1 and BNT162b2), and many countries along with the European Union authorized its use in December 2020 [17]. The United Arab Emirates and Bahrain granted marketing access to the Sinopharm BIBP vaccine [18]. The mRNA-1273, the Moderna Vaccine, was also approved by the FDA in the same month [19]. Oxford University in collaboration with British Swedish Company AstraZeneca developed another vaccine using a vector, the modified chimpanzee adenovirus ChAdOx1, which was named Covishield [20].

India’s population of 1.39 billion posed a challenge for its government, yet it launched the world’s largest vaccine drive on January 16, 2021 [21]. Two vaccines were made available initially, namely, Covishield (ChAdOx1 nCoV-19_Oxford-AstraZeneca) manufactured by the Serum Institute of India, and Covaxin (BBV152) by Bharat Biotech. Later, the other Sputnik V (Gam-COVID-Vac) made by Gamaleya Research Institute of Epidemiology and Microbiology, Moderna COVID-19 (mRNA- 1273) by the US FDA were also introduced. Covishield is the recombinant chimpanzee adenovirus vector encoding SARS-CoV-2 spike (S) glycoprotein. On administration, the genetic material is expressed which stimulates the immune response. The regime consisted of two doses, given 12-16 weeks apart. Covaxin, India’s own COVID-19 Vaccine, is a whole virion-inactivated Vero Cell developed by Bharat Biotech in collaboration with the Indian Council of Medical Research (ICMR)-National Institute of Virology (NIV). Sputnik V was developed on the human adenovirus vector platform and was so named after the first Soviet space satellite. Moderna COVID-19 vaccine, brought in by an American company named Moderna in association with the US National Institute of Allergy and Infectious Diseases (NAID) and the Biomedical Advanced Research and Development Authority (BARDA), is an mRNA vaccine made of nucleoside-modified mRNA encoding a spike protein of SARS-CoV-2, which is encapsulated in lipid nanoparticles [22].

Although the SARS-CoV-2 immunity varies and associations remain fairly elusive, the role of both T-cell and B-cell in protection against the infection is now standardized. ChAdOx1 nCoV-19, through the S-protein gene enveloped inside the adenovirus vector, channels a reverse transcription in the host cells, leading to the generation of S-protein spiked cells in the body. These spikes are recognized by the antigen-presenting cells, in turn, inducing a strong and extensive T-cell response in the host which includes majorly a Th1 stimulation leading to the synthesis of interferon-gamma, interleukin-2 and tumor necrosis factor-α/β. The interferon endorses the differentiation of Cd4+ and CD8+ effector T-cells and CD4+ T follicular helper cells that induces B-cell transformation and proliferation, converting them to plasma cells secreting anti-IgA and anti-IgG antibodies to the coronavirus S-protein [23-25]. The mechanism of immune response for Covaxin is similar except that the inactive virus contains the active S-protein. When these inactive virus particles are picked up and processed by host APCs, the fragments with the S-proteins are detected by the helper T-cells followed by their activation to initiate the rest of the immune process [26-28]. These antibodies that are released in reaction to the invasion are called NAbs. This study aimed to detect and measure these NAbs to assess the efficacy and strength of the protection of these vaccines.

Since the first phase of vaccination, numerous studies reported their efficacy results from different vaccination centers and cohorts globally. While different countries had different vaccines marketed in them, the ultimate response was similar in the studies. The present study assessed the effectiveness of Covishield and Covaxin and found that the medians levels of the NAbs rose to their peak after the second dose. This was true for all other studies (Table 6) [29-38]; however, the fall in the levels of the antibodies on further assessment as reported by various studies could not be compared yet.

**Table 6:** Studies reported from across the globe HCW: healthcare worker; BMI: body mass index; NAb: neutralizing antibody

Title of paper	Authors	Site of study	Cohort	Vaccines	Remark
Analysis and comparison of anti-RBD neutralizing antibodies from AZD-1222, Sputnik V, Sinopharm and Covaxin vaccines and its relationship with gender among health care workers	Zare et al. [29]	Iran	HCW	AZD-1222, Sputnik V, Sinopharm, Covaxin	Sputnik V and AZD-1222 were reported to be more effective than Sinopharm and Covaxin. The vaccine efficacy was irrespective of the gender
Anti-SARS-CoV-2 antibody responses 5 months post complete vaccination of Moroccan healthcare workers	Assaid et al. [30]	Morocco	HCW	ChAdOx1 nCoV-19 BBIBP-CorV	Similar levels of antibody titers were reached after vaccination with either of the vaccines, and the titers were independent of age and gender
Comparison of SARS-CoV-2 antibody response following vaccination with BNT162b2 and mRNA-1273	Steensels et al. [31]	Belgium	HCW	mRNA-1273 BNT162b2	Antibody titers were higher in participants vaccinated with two doses of mRNA-1273 compared with those vaccinated with BNT162b2. They also found a negative correlation between age and antibody titer
Humoral immune response after COVID‑19 infection or BNT162b2 vaccine among older adults: evolution over time and protective thresholds	Meyer et al. [32]	France	Elderly volunteers	BNT162b2	The vaccine response lasted till the sixth month of the vaccination. The antibody titer was higher in the males but the difference was not statistically different
Dynamics of antibody response to BNT162b2 vaccine after six months: a longitudinal prospective study	Naaber et al. [33]	Estonia	Healthy volunteers	BNT162b2	The antibody response to the spike protein peaked after the second dose but started to decline after 12 weeks of vaccination
Anti-SARS-CoV-2 spike protein RBD antibody levels after receiving a second dose of ChAdOx1 nCov-19 (AZD1222) vaccine in healthcare workers: lack of association with age, sex, obesity, and adverse reactions	Lee et al. [34]	Japan	HCW	ChAdOx1 nCoV-19	After the second dose, 100% seropositivity was observed. Antibody response was independent of age, sex, and BMI
Evaluation of anti-SARS-Cov-2 S-RBD IgG antibodies after COVID-19 mRNA BNT162b2 Vaccine	Lo Sasso et al. [35]	Italy	Volunteers	BNT162b2	IgG levels were decreased within a short span of time. Anti-RBD antibody IgG levels were higher in females. No difference was found between previously infected and non-infected groups
Lasting SARS-CoV-2 specific IgG antibody response in health care workers from Venezuela, 6 months after vaccination with Sputnik V	Claro et al. [36]	Venezuela	Volunteers	Sputnik V	The antibody levels dropped to 50% between the 42^nd^ and 180^th^-day post-vaccination. A seropositivity of 94% was observed after six months
Comparison of the effectiveness and duration of anti-RBD SARS-CoV-2 IgG antibody response between different types of vaccines: implications for vaccine strategies	Sughayer et al. [37]	Jordan	Blood bank donors and HCW	Pfizer/BioNTech, AstraZeneca, Sputnik V, Johnson & Johnson, Moderna, and Sinopharm	The highest mean levels of anti-RBD antibody titer were seen with the Pfizer group, which decreased significantly by the 60^th^ day of vaccination. Though the AstraZeneca and Sinopharm, did not induce a very high antibody titer, the levels the maintained till the 120^th^ day of vaccination
Diferential persistence of neutralizing antibody against SARS‑CoV‑2 in post immunized Bangladeshi population	Roy et al. [38]	Bangladesh	Healthy adults	CoviShield (AZD1222)	While 42.9% of the subjects showed a more than 96% rise in NAb titer 30 days after the vaccination, only 5.1% retained the levels at the 180^th. ^Only previously infected volunteers maintained the antibody titers even after 180 days of vaccination

Although the overall seroconversion rate of 76.67% was lower than that of other studies done in India, the higher efficacy of Covishield (100% seroconversion) was true for all studies including the present study (Table 7) [26-28,39].

**Table 7:** Studies from in India. HCW: healthcare worker

Title of paper	Authors	Site of study	Cohort	Vaccines	Remark
Persistence of antibodies against spike glycoprotein of SARS-CoV-2 in healthcare workers post double dose of BBV-152 and AZD1222 vaccines	Choudhary et al. [26]	Bhuvaneshwar	HCW	BBV-152 AZD1222	The levels of post-vaccine IgG antibodies were significantly higher (p < 0.001) in Covishield administration than with Covaxin. The former group produced higher anti-S IgG titer too. Age, gender, comorbidities, and blood groups did not affect the levels significantly
Antibody profile in post-vaccinated & SARS-CoV-2 infected individuals	Patil et al. [27]	Mumbai	Volunteers	Covaxin (BBV152) Covishield (ChAdOx1 nCoV-19)	An overall seropositivity of 97.7% was observed in all vaccinated individuals. Covishield was more potent in generating neutralizing antibodies, and even a single dose was effective
Study of neutralizing (anti-RBD) antibody responses induced by COVID-19 vaccines in healthcare professionals in a diagnostic centre of central India	Hawaldar et al. [39]	Indore, MP	HCW	Covishield	Seroconversion rate was 88.24% after about a month from the first dose, and 96.0% conversion was observed after the booster dose. The level of the neutralizing antibody remained high on the 68^th^ day of vaccination
Antibody Response after first-dose of ChAdOx1-nCOV (CovishieldTM) and BBV-152 (CovaxinTM) amongst health care workers in India: preliminary results of cross-sectional coronavirus vaccine-induced antibody titre (COVAT) study	Singh et al. [28]	Multicentric	HCW	Covishield Covaxin	The median levels of anti-spike antibody and the rate of seropositivity were higher in the Covishield group, though both the vaccines induced good immune response

This study also found a higher median level of the anti-RBD antibodies in the volunteers vaccinated with Covishield, which was similar to the findings of studies conducted by Choudhary et al. [26], Patil et al. [27], and Singh et al. [40]. The clinical-demographical variables compared in this study showed no significant effect on the antibody response and vaccine efficacy. This result was at par with studies done by Choudhary et al. [26], Salvagno et al. [41], Assaid et al. [30], and Lee et al. [34]. This study and the study conducted by Lo Sasso et al. [35] found that the levels of antibodies did not differ significantly between the previously infected and non-infected groups.

This study was the first pilot study from the state of Chhattisgarh and contributes effectively to the prominent question of which vaccine is better. However, the study had its limitations such as a small sample size due to fewer patients reporting back for the second dose. The follow-up of these patients could not be done and the post-vaccination infection was not analyzed. Thus, a further study for the above with a larger sample size would provide better answers.

## Conclusions

The present study was the first of its type in this population to analyze the efficacy of the two available vaccines in the state, as implemented by the government. Although Covishield was a better vaccine in terms of circulating antibodies, seropositivity, and adverse effects, both vaccines could be used effectively to fight another outbreak of COVID-19. In a country like India, where mass vaccination is needed, a self-made vaccine could be the answer to decrease morbidity and mortality.
